# Why the need and how to approach the functional diversity of extracellular vesicles

**DOI:** 10.1098/rstb.2016.0479

**Published:** 2017-11-20

**Authors:** Mercedes Tkach, Joanna Kowal, Clotilde Théry

**Affiliations:** Institut Curie, PSL Research University, INSERM U932, 26 rue d'Ulm, 75005 Paris, France

**Keywords:** extracellular vesicles, exosomes, multi-vesicular endosomes, cancer

## Abstract

In the past decade, cell-to-cell communication mediated by exosomes has attracted growing attention from biomedical scientists and physicians, leading to several recent publications in top-tier journals. Exosomes are generally defined as secreted membrane vesicles, or extracellular vesicles (EVs), corresponding to the intraluminal vesicles of late endosomal compartments, which are secreted upon fusion of multi-vesicular endosomes with the cell's plasma membrane. Cells, however, were shown to release other types of EVs, for instance, by direct budding off their plasma membrane. Some of these EVs share with exosomes major biophysical and biochemical characteristics, such as size, density and membrane orientation, which impose difficulties in their efficient separation. Despite frequent claims in the literature, whether exosomes really display more important patho/physiological functions, or are endowed with higher potential as diagnostic or therapeutic tools than other EVs, is not yet convincingly demonstrated. In this opinion article, we describe reasons for this lack of precision knowledge in the current stage of the EV field, we review recently described approaches to overcome these caveats, and we propose ways to improve our knowledge on the respective functions of distinct EVs, which will be crucial for future development of well-designed EV-based clinical applications.

This article is part of the discussion meeting issue ‘Extracellular vesicles and the tumour microenvironment’.

## 1. Introduction

The description of membrane-enclosed structures, smaller than mammalian cells and found in the extracellular space, began in the late 1960s. They were observed by researchers describing coagulation- or calcification-inducing factors in, respectively, plasma or bones. Names then chosen to design what we now call extracellular vesicles (EVs) were ‘platelet-dust’ [1], or matrix vesicles [2]. In the 1970s and 1980s, terms like microparticles [3], microvesicles [4,5], ‘membrane fragments’ [6] and ‘membrane vesicles’ [7,8] started being used to describe vesicles generated by cultured tumoural and non-tumoural cell lines, or recovered in biological fluids. In these articles, vesicles were shown to bear pro-coagulant or enzymatic activities, and thought to be shed from the cells’ plasma membrane. In 1983–1985, the groups of Stahl and of Johnstone demonstrated that cells could also secrete membrane vesicles by a three-step process of endocytosis, formation of intraluminal vesicles (ILVs) inside endosomes and fusion of the multi-vesicular endosomes (MVE) with the plasma membrane, which results in release of the ILVs [7,9]. Reticulocytes were shown to secrete such MVE-derived EVs to eliminate unnecessary transmembrane proteins. The term ‘exosomes’ was proposed in 1987 to refer to this specific type of EVs [10], although two articles had previously used it for ‘exfoliated’ membrane vesicles presumably formed at the plasma membrane [5,11]. After 1987, the word exosomes started being used specifically for EVs of endosomal (MVE), rather than plasma membrane, origin. In the late 1990s, two groups working on antigen-presenting cells of the immune system [12,13] showed that exosomes also carried surface molecules that could induce signalling in target cells, hence that they were not only ‘trash cans’, but also inter-cellular communication devices. This discovery renewed the interest of biomedical scientists in exosomes, and even though microvesicles and other shed membrane vesicles had been studied for their communication functions for more than three decades, the term ‘exosomes’ started outnumbering the term ‘microvesicles’ in the scientific literature in 2002. Since then this has become the most frequently used term in the EV field (see fig. 1 of [14]). This fact somehow conveys to non-specialists the impression that exosomes are more interesting or more biologically important than other EVs. The point we want to make in this short article is that our current knowledge on the respective functions of exosomes and other EVs is too partial to either confirm or contradict such an assumption. Consequently, despite the current massive effort of biotech and biopharmaceutical companies to develop exosome-based clinical tools, we cannot knowingly decide whether exosomes are the best choice as therapeutic targets or diagnostic analytes. We think that the field must now evolve to systematically and comprehensively compare the nature and the functions of all secreted EVs. This knowledge will make it possible to identify, on the one hand, molecular components and activities shared between several (or all) EV subtypes, and on the other hand, the components and activities specific to one subtype of EVs, which could be, for instance, MVE-derived exosomes, but also non-exosomal EVs.

## 2. Mixed nature of exosome and extracellular vesicle preparations

As exosomes are formed as ILVs of MVE, they present a diameter of 50–150 nm, similar to the diameter of ILVs observed by electron microscopy (see examples and review on the endosomal pathway in [15]). However, this restricted size is necessary, but not sufficient, to define exosomes. Indeed, EVs budding from the plasma membrane do not present a limited size distribution, which means that they can be much larger (up to one or a few micrometres in diameter), but also as small as exosomes (smaller than 150 nm in diameter). Most protocols used to isolate exosomes are designed to isolate small EVs and eliminate larger ones, by, for example, filtration through 0.22 µm filters, or by differential ultracentrifugation (dUC) whereby large/heavy vesicles are first pelleted and eliminated at low/medium speed [16–18]. The resulting samples, even if they are generally called ‘exosomes’, in fact potentially contain a mixture of small EVs, whose subcellular origin can be heterogeneous. This idea first occurred to us when we realized that, upon inhibiting expression of the small GTPase Rab27a in a murine tumour cell line [19], we observed a decreased secretion of some exosome-associated proteins, such as the tetraspanin CD63, TSG101, HSC70 (gene name: *Hspa8*), as described in a human HeLa cell line [20]), but not of others, which were so far considered also as ‘exosome-markers’, such as another tetraspanin, CD9, or the phosphatidylserine-binding protein Milk Fat Globule-EGF-Factor VIII (MFGE8). As CD63 and TSG101, in the secreting cell, accumulate in MVE, whereas CD9 is found at or just below the plasma membrane [19], this observation suggested that small EVs originating from both MVE and plasma membrane or another subcellular compartment were co-isolated in ‘exosome’ preparations. As Rab27a inhibition leads to a partial inhibitory effect on *in vivo* growth of this tumour cell line, and no effect on another murine tumour cell line [21], we speculate that the Rab27a-independent small EV population may display some functions in tumour growth that remain to be elucidated. In other cells or experimental systems, different intracellular machineries required for ‘exosome’ formation and secretion have been described, such as other Rab GTPases, or endosomal sorting complex required for transport (ESCRT)-dependent or -independent mechanisms of biogenesis (reviewed in [22]). As different EV-associated proteins are used in different studies to characterize the analysed EVs, it is likely that these different machineries are involved in formation of different subtypes of EVs [23]. Until last year, several proteins were equivalently used to qualify EVs as exosomes, based on their previous identification by proteomic studies [24]. For the majority of these ‘exosome markers’, no demonstration of the specificity for EVs of MVE origin, when compared with EVs from any other subcellular location, had been provided. The field, however, has progressed, and based on recent comparative proteomic analyses, we can now begin proposing a list of protein markers qualifying some subtypes of EVs.

## 3. Recent advances in distinguishing extracellular vesicle subtypes

Until five years ago, proteomic studies investigating the content of EVs were generally focused on small EVs recovered by dUC and/or filtration [24]. Owing to limited interest in different EV subtypes, they provided a crude list of proteins present in small EVs, without any comparative or quantitative evaluation of these proteins in other types of EVs. We thus decided to compare side-by-side distinct EVs, in order to better understand the nature of isolated EVs and to develop tools to describe and distinguish distinct EV subtypes.

**Figure 1.**
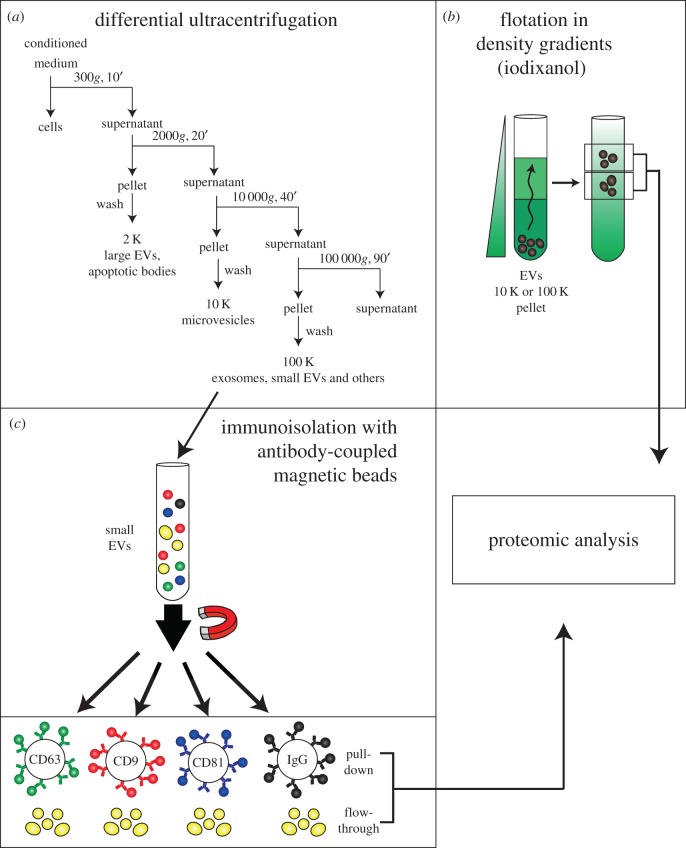
Different approaches to analyse the heterogeneity of EVs (based on [26]). (*a*) Scheme of EV isolation by dUC protocol from a mixture of EVs present in cell culture conditioned medium or body fluids. For the latter EV source, additional steps of biofluid processing before centrifugation are recommended to eliminate abundant EV-excluded components. (*b*) Further separation of the 10 K and 100 K pellets obtained by the dUC protocol through flotation in a density gradient (iodixanol) allowed us to distinguish discrete populations of EVs by proteomic analysis. (*c*) Separation of subtypes of small EVs through immunoisolation. We isolated small EVs bound specifically to beads coupled to antibodies to CD9, or to CD63, or to CD81 or to irrelevant IgG as control. We analysed these EV subpopulations by proteomic analysis in parallel with the non-pulled down materials remaining in the flow-through for each immunoisolation. Comparing EVs pulled down via the different tetraspanin-specific antibodies identified an exosomal EV subpopulation as bearing CD63 together with the other tetraspanins. Analysing the flow-through demonstrated the presence of non-exosomal small EVs in the 100 K pellet.

We focused on human primary dendritic cells (DCs), which we had shown to secrete more heterogeneous small EVs than, for instance, the HeLa tumour cell line [25]. We used different approaches to separate EV subtypes. The first approach was based on the different sizes and physical properties of EVs, resulting in different pelleting behaviours and different flotation into density gradients. The second approach was based on EVs' surface composition, resulting in differential isolation by antibody-based capture (figure 1) [26]. As proposed before [16], increasing the speed of centrifugation resulted in EV preparations progressively enriched in EVs of decreasing sizes. A majority of EVs of diameter larger than 200 nm were recovered upon centrifugation at 2000*g*. A mixture of large, medium size and small EVs was obtained at 10 000*g*. Finally, the smallest EVs (less than 150 nm) formed the vast majority of the pellet after 100 000*g* ultracentrifugation. We analysed the proteome of four types of vesicles that were pelleted at different speeds (10 000*g* and 100 000*g*) and reached different buoyant densities (1.115 and 1.145 g ml^−1^) after bottom–up flotation into a density iodixanol (= Optiprep™) gradient. By gene ontology term analysis of the qualitative proteome of each fraction, we showed that the denser fraction (1.145 g ml^−1^) of the 100 000*g* pellet was enriched in extracellular matrix (fibronectin, collagen) and serum-derived proteins (albumin, complement), but not in late endosome-associated proteins. On the contrary, the lighter fraction of the same pellet was the only fraction presenting enrichment in both plasma membrane and late-endosomal proteins, thus the only fraction containing exosomes (in addition to other small EVs). Mitochondrial, endoplasmic reticulum, ribosomal and proteasomal components were, by contrast, mostly present in one or the other fraction of the 10 000*g* pelleted EVs, which therefore came from different intracellular compartments than small EVs. Importantly, we could identify proteins that are common to all analysed EV subpopulations. These proteins include: cytoskeletal proteins (actin, ezrin, moesin), heat shock protein 70, flotillin-1 and major histocompatibility complex class I and II molecules, among others. We thus propose that these proteins could be defined as ‘core’ components of any given EV, especially because we confirmed their abundance in EVs from many different cell lines. The presence of these proteins in any given preparation of EV will not indicate whether one is analysing EVs from plasma membrane, from MVE (exosomes) or from any other subcellular origin, but will confirm the presence of EVs in the preparation.

On the other hand, we could define some proteins that were exclusively present in one and not the other type of EVs, and shared proteins displaying different abundances in different EV types; for instance, proteins specific to small exosome-like EVs that are excluded from large EVs and vice versa. As examples, cytoskeletal actinin-4 (gene name *ACTN4*), mitochondrial inner membrane mitofilin (*IMMT*), endoplasmic reticulum GP96 (=endoplasmin, *HSP90B1*) and major vault protein MVP are present in large EVs with medium size greater than 200 nm. On the other hand, proteins mainly secreted in small EVs of diameter smaller than 150 nm include TSG101, syntenin-1 (*SDCBP*), ADAM10 and CD81. In addition, we also showed that among these small EV-associated proteins, a few were specific of late endosome-derived exosomes, whereas many others were also present in other subtypes of small EVs. To reach this conclusion, we developed an immunoprecipitation assay in which we pulled down the vesicles recovered in the 100 000*g* pellet by antibodies specific to the three members of the tetraspanin family classically used as exosome markers (CD63, CD9 and CD81) and simultaneously analysed the vesicles negative for these tetraspanins that were not bound to antibody-covered beads (flow-through, figure 1). Mass spectrometry analyses showed that the preparation of small EVs with exosome-like morphology by EM is indeed a mixture of heterogeneous EVs. We observed an enrichment of late endosome proteins in the subtype of EVs pulled down by anti-CD63, and containing also CD9 and CD81, but not in EVs that did not contain CD63, but displayed only CD9, and/or CD81, or none of these tetraspanins. TSG101 and syntenin-1, and a few other proteins involved in transmembrane receptor targeting to ILVs of MVEs and secretion of MVE-derived EVs [25,27], were exclusively recovered in this subtype of small EVs, which thus probably corresponds to *bona fide* exosomes. It is important to note that single presence of CD63 does not qualify an EV as exosome, because CD63 was also detected in large EVs. Similarly, although CD81 was exclusively detected in small EVs, its single presence without CD63 does not qualify a small EV as exosome, because CD81+/CD63− EVs did not present an enrichment in MVE-derived proteins. Finally, we also detected small EVs containing CD9 only, or vesicles that were not bearing any of these tetraspanins, but were still positive for other transmembrane proteins (e.g. MHC molecules or integrins), which likely form at the plasma membrane or in early and/or recycling endosomes. A summary of these results is provided in table 1.

**Table 1.** Distribution of proteins identified by comparative quantitative proteomic (MS) in different subtypes of EVs isolated from human monocyte-derived DCs [26]. Validation by western blot (WB) was performed for most of them on large (2 K pellets), medium (10 K pellets) and small EVs (100 K pellets) isolated from DCs and the listed established human cell lines, or mouse bone marrow-derived DCs (BMDCs). Information on antibodies used for WB is provided in Kowal *et al.* [26]. Proteins highlighted in bold are those identified by immunoisolation as probably specific to MVE-derived exosomes, bearing simultaneously CD63 and CD9 or CD81.

protein name/*gene/*UNIPROT ID	vesicle type	detection method	cell type
actin cytoplasmic/*ACTB, ACTG1/*ACTB, ACTG	all EVs	MS, WB	DCs
annexin II or A2/*ANXA2/*ANXA2	all EVs	MS, WB	DCs
flotillin-1/*FLOT1*/FLOT1	all EVs	MS, WB	DCs
HSC70/*HSPA8*/HSPC7	all EVs	MS, WB	DCs, MDA-MB-231, IGROV, OV2008, SHIN, HeLa, HEK, RPE1, mouse BMDCs
HLA (MHC) class I/*HLA-A, HLA-B, HLA-C/*1A*, 1B*, 1C*	all EVs	MS, WB	DCs
HLA (MHC) class II/HLA-DR**, HLA-DP**, HLA-DQ**/2B**, DP*, DQ*, DR*	all EVs	MS, WB	DCs, mouse BMDCs
alpha-actinin-4/*ACTN4/*ACTN4	large EVs	MS, WB	DCs, MDA-MB-231, IGROV, OV2008, SHIN, mouse BMDCs
GP96 = endoplasmin/*HSP90B1*/ENPL	large EVs	MS, WB	DCs, MDA-MB-231, IGROV, OV2008, SHIN, mouse BMDCs
lysosome-associated membrane glycoprotein 2/*LAMP2*/LAMP2	large EVs	MS, WB	DCs
mitofilin/*IMMT* /IMMT, MIC60	large EVs	MS, WB	DCs
annexin XI or A11/*ANXA11/*ANX11	small EVs	MS, WB	DCs
disintegrin and metalloproteinase domain-containing protein 10/*ADAM10*/ADA10	small EVs	MS, WB	DCs, MDA-MB-231, IGROV, OV2008, SHIN, HEK, RPE1
EH domain-containing protein 4/*EHD4*/EHD4	small EVs	MS, WB	DCs
fibronectin/*FN1*/FINC	dense small EVs	MS	DCs
**CD9 (tetraspanin)/*CD9*/CD9**	**abundant in small EVs but also present in large EVs**	**MS, WB**	**DCs, MDA-MB-231, IGROV, OV2008, SHIN, HeLa, HEK, RPE1, mouse BMDCs**
**CD63 (tetraspanin)/*CD63*/CD63**	**in small EVs: specific for exosomes. Also present in large EVs**	**MS, WB**	**DCs, MDA-MB-231, OV2008, HeLa, HEK, RPE1, mouse BMDCs**
**CD81 (tetraspanin)/*CD81*/CD81**	**small EVs**	**MS, WB**	**DCs, MDA-MB-231, IGROV, OV2008, SHIN, HeLa, HEK, RPE1**
**TSG101/*TSG101*/TS101**	**small EVs**	**MS, WB**	**DCs, mouse BMDCs**
**syntenin-1/*SDCBP*/SDCB1**	**small EVs**	**MS, WB**	**DCs, MDA-MB-231, IGROV, OV2008, SHIN, HeLa, HEK, RPE1, mouse BMDCs**

Our study is the most extensive so far, in terms of numbers of EV subtypes analysed, but other studies have also compared the protein composition of a limited number of EV subtypes (generally two) recovered either at intermediate versus high-speed centrifugation and/or after density gradient separation [28–33], or by immunoisolation with anti-A33 or anti-EpCAM antibodies [34]. Comparison of the latter study with our own results suggests that the A33-positive small EVs, which do not bear CD63/CD81 and contain little syntenin, would not qualify as *bona fide* exosomes, whereas the EpCam-positive small EVs would. This study thus confirms the simultaneous presence of both exosomal and non-exosomal EVs in the high-speed dUC pellets.

## 4. Current need and how to determine if all extracellular vesicles and/or exosomes have the same functions

**Figure 2.**
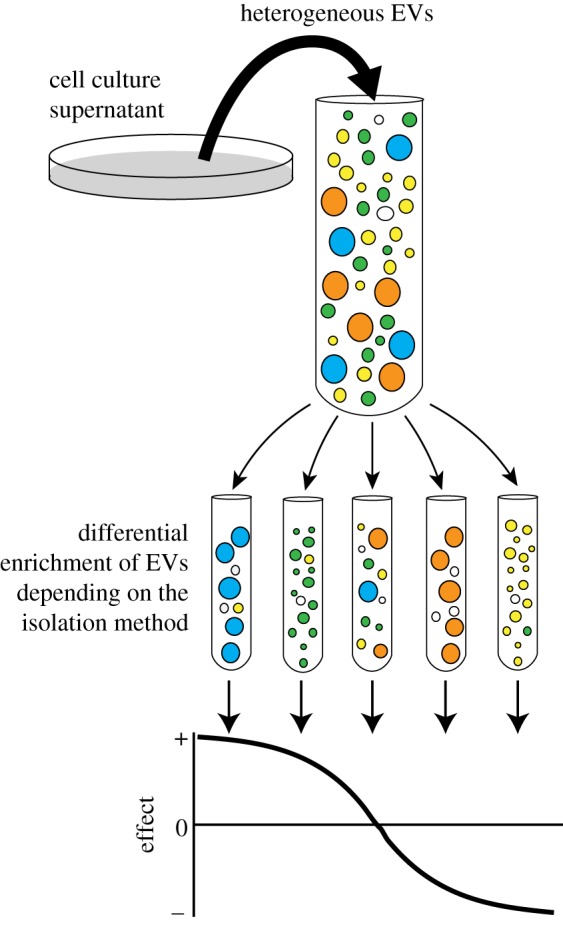
Functional analysis of heterogeneous EVs populations. When analysing the functionality of EVs released by a cell line, a primary culture or even from body fluids, the results can be extremely biased depending on the isolation technique used. For example, in the scheme, we illustrate a case where both large blue EVs and small green EVs have a positive effect on the analysed target cells, while the large orange EVs and small yellow EVs have a negative effect. Depending of the ratio of these EVs in a given preparation, we can either observe a positive or a negative effect, or even no effect if the mixture of all EV subtypes compensates the respective effects.

Given the inefficacy of the current exosome isolation protocols to provide pure populations of a given EV subtype, all studies analyse the functions of mixtures of EVs. Depending on the strategy applied for isolation, EV preparations are either a heterogeneous population of small EVs or mixtures of EVs of all sizes when the step of elimination of large EVs is omitted (for instance, by protocols involving direct concentration of the conditioned medium by ultracentrifugation or polymer-based precipitation). Knowing this limitation of the so far published studies, we cannot anymore assume that only exosomes, and no other small or large EVs, display particularly important physiological or pathological functions, making them a desired specific therapeutic target or vector, for the reasons detailed in the following paragraphs (figure 2).

On the one hand, in a preparation of small EVs containing MVE-derived vesicles and vesicles that had formed at the plasma membrane, the presence of specific components (of proteic, lipidic or nucleic acid nature) in these different subtypes of EVs can result in different functional properties. If two subpopulations display opposite effects on a particular analysed function, the final effect is dependent on the respective proportions of each EV. Alternatively, one major subpopulation may not bear the desired activity, but still be the one quantified and characterized to validate the quality of the EV preparation. Consequently, an actually important function of a given EV subtype may be dampened or masked by the presence of the other subpopulation. A pure population of the EV subtype displaying the positive activity would bear tremendously increased effect and reproducibility, and thus represent the actual target of choice for future therapeutic applications.

Conversely, if the analysed function is equally displayed by all types of EVs—in other words, not only exosomes but small EVs of all subcellular origins and also larger EVs that are rarely analysed—trying to specifically inhibit the secretion of exosomes or small EVs to prevent their detrimental function (like prometastatic ability) for therapeutic intervention will be poorly effective as all the remaining disregarded EVs will remain present and achieve this activity. For a favourable activity of EVs, which could be exploited for therapeutic purposes (like activation of immune responses), if this activity is shared by all subtypes then there would be no need to go into complicated separation methodologies when a cruder isolation process would recover larger amounts of active EVs.

We thus think that comparing side-by-side the activities of as many EV subtypes as can possibly be separated by the currently available protocols represents a crucial step that must now be taken in all functional studies of EVs. A few groups started performing these kinds of approaches in the past few years. For instance, Keerthikumar *et al*. [28] observed a stronger proliferative and pro-migratory effect of small EVs (containing exosomes) than large EVs (called ectosomes) secreted by neuroblastoma cell lines, both populations being separated by pelleting into an iodixanol gradient. Conversely, Minciacchi *et al*. [35] showed more efficient fibroblast reprogramming and endothelial cell tube formation by large oncosomes, when compared with the small EVs secreted by the same prostate cancer cell lines. We compared the T lymphocyte-activating potential of EV subtypes secreted by human DCs [36], separated by dUC and flotation into iodixanol (as performed in [26]). Importantly, we observed that all EVs, large, medium and small, were efficiently able to present allogeneic MHC-peptide complexes to CD4^+^ helper T cells: thus, T cell activation is a shared property of all DC-derived EVs. However, the different subtypes of EVs induced different patterns of polarization of activated T cells. Large EVs secreted by immature DCs promoted secretion of Th2-associated cytokines, whereas both medium and small EVs (10 000*g* and 100 000*g* pellets) activated secretion of Th1 cytokines. When isolated from mature DCs, all EV pellets induced mainly Th1 cytokines. Unexpectedly, upon flotation into iodixanol gradient, the exosome-enriched lighter fraction was proportionally less efficient at inducing Th1 cytokines than the denser vesicle fraction enriched in extracellular matrix proteins. It suggests that the Th1-inducing activity is not a major feature of *bona fide* exosomes. Therefore, qualitative functional activities of different EVs are worth exploring in detail, and it is crucial to clearly unravel the specific functions of MVE-derived exosomes versus non-exosomal small EVs. We are, however, still lacking separation techniques that can be used for downstream functional assays. Although using immunoisolation of the different small EVs by anti-CD63 antibodies should make it possible to recover the purest possible exosome population, the remaining antibody and/or beads used for purification in the exosome preparation prevent accurate functional studies, because they are likely to modify interaction of EVs with their target cells. Novel means of small EV subtype separations, or techniques to clean EVs from the isolating molecules, will have to be developed.

Secretion of large EVs containing intact intracellular organelles or active cytoskeleton has been described for tumours [37–39], and proposed to play a role in tumour migration or interaction with the immune system. The complex nature of these large EVs suggests that their functions could be even more diverse than those of exosomes or small EVs. However, obtaining pure preparations of these large EVs may be challenging: the low-speed centrifugation pellets also contain small EVs [26], and possibly whole cells, which, even though present in very low numbers, could affect the read-out of functional tests. Thus, care to use proper controls is an important aspect of analysing the functions of large EVs.

## 5. How to quantify extracellular vesicles for functional assays

Finally, an important question to consider when quantitatively investigating the functions of EV subtypes or EVs from different sources is how to normalize the different samples to compare. First, quantifying EV preparations is not as straightforward as it seems. The total amount of proteins present in an EV preparation, measured with a sensitive and miniaturized colorimetric method such as micro bicinchoninic acid, is often used. One potential pitfall is that the apparent concentration of EV preparations not sufficiently cleaned from co-isolated protein contaminants (such as albumin from the fetal calf serum used for culture, or other abundant proteins from biological fluids) may be artificially overestimated. Over the past decade, a few devices designed to count and size particles of nanometric sizes that are difficult to analyse reliably with regular flow cytometers or by fluorescent microscopy have been developed by several companies. One of the major devices currently used [17] is the nanoparticle tracking analyzer (NTA) from Malvern (formerly Nanosight). The NTA analyses Brownian motion of particles illuminated by a laser, from which it deduces their size and calculates their concentration. A similar device called Zetasizer has been recently developed by another company (Particle Metrix), and a slightly different method based on dynamic light scattering is less commonly used. Another principle called tunable resistive pulse sensing (TRPS) is used by the qNano device (Izon): it measures blockade of impedance of a nanopore when individual particles go through it, and calculates the amount and size of the particles. All these devices calculate the number of particles present per volume of sample, but they do not really provide absolute numbers that could be reproducibly compared between different laboratories and users. This is because these devices require some manual settings, which can be to some extent user-dependent. Recently, efforts to standardize and to compare results between different laboratories have been published for NTA [40] and TRPS [41]. Importantly also, none of these devices is able to distinguish membrane-enclosed vesicles from non-vesicular particles of the same size. Hence, the actual concentration eventually obtained is not specific to EVs, because it also counts other particulate structures. Finally, quantifying lipids in EV preparations is another interesting possibility, because it takes into account the defining component of EVs: the lipid bilayer that defines them as vesicles. Such a quantification assay has been recently published [42]. However, because its sensitivity is not very high, characterizing an EV preparation with this assay uses, in our experience, too much of the sample so that it is difficult to keep enough for functional assays. Therefore, so far, combining quantification of total proteins and particle number is still the best way to quantify materials present in an EV preparation.

This information can be used to equalize amounts of different EVs used for a functional assay, and published articles generally use either protein amount or the particle number to do so, but not both. This approach is valid if both criteria change similarly between the two analysed EV preparations. However, when we tried to use these criteria to compare the functions of large EVs pelleted at low speed, versus small EVs pelleted at high speed, we faced the following problem: large EV-enriched samples generally contained more proteins, but fewer particles than small EV-enriched samples [26,36]. Therefore, choosing protein amount to normalize the amount of each EV type used in the functional assay would mean using a smaller volume of large than small EVs, whereas choosing particle number would mean using a larger volume of large than small EVs. In either case, the final read-out would be biased in opposite manners. One way to overcome this difficulty is to normalize samples by the amount of EV pellet secreted by a given number of EV-donor cells, meaning EVs present in a given volume of conditioned medium [36]. This choice also has the advantage of mimicking the physiological situation where a target cell encounters all EVs released simultaneously by the secreting cells. Normalization by number of EV-secreting cells was used by Lo Cicero *et al.* [43] to show the effect of small EVs released by keratinocytes on melanocytes. In this work, an interesting feature is that the authors used a 1 : 1 ratio of EV-donor to EV-recipient cells, not very far from the respective proportions of keratinocytes and melanocytes present in the skin. The authors also analysed the activity of conditioned medium depleted of small EVs, when compared with that of isolated EVs, and observed that some functional outcomes were present in the soluble portion of the conditioned medium. It would therefore be advisable to perform such comparisons and controls for any functional analysis of EVs, to convincingly document the actual physiological relevance of EV secretion.

## 6. Conclusion

We hope that we have convinced the reader of the need to compare the different possible EVs before concluding on the specific involvement of one EV type, for instance, exosomes, in a given function. Ways to obtain pure EV subtypes, or at least to determine the relative proportion of different EVs in a given sample, are not yet fully determined, although progress has recently been made. With increasing numbers of comparative proteomic studies of EV subtypes, we may be able in the near future to propose protein markers defining the different types of EVs that will be valid to analyse EVs from all possible cellular or biological fluids. In the meantime, we propose that EVs isolated by 100 000*g* dUC or any similar protocol should be called small EVs, rather than ‘exosomes’ until the necessary steps to prove their endosomal origin, or to separate the endosomal EVs from the others, have been performed. On the other hand, analysing the functions of large EVs, when compared with those of small EVs, may not be necessary for some EV sources. Indeed, whereas primary DCs of both mouse and human origin release a lot of large EVs recovered by low-speed centrifugation, for several tumoural and non-tumoural cell lines, we recovered little material in the pellet enriched in large EVs [26]. For such cell sources, therefore, further analysis of the functions of large EVs may not be useful. But before reaching this conclusion, it is important to first perform the dUC and successive pellet analysis.

Importantly, one of the most highlighted functions of EVs in the past decade is their ability to transfer mRNA and miRNA to target cells, and thus deeply modify their behaviour by affecting gene expression [23]. For this type of activity, however, unravelling the heterogeneity of EV subtypes and their nucleic acid cargo is an even more crucial need that is as yet unfulfilled. Indeed, description of the presence of RNAs in EVs initiated the idea that RNAs can have an extracellular function [44,45], and this concept was soon expanded by reports showing the presence in biofluids of extracellular RNA in non-vesicular carriers, such as ribonucleoprotein complexes or lipoproteins [46,47]. A quantitative stoichiometric study then demonstrated that a given miRNA molecule is present at far less than one molecule per particle in a high-speed ultracentrifugation ‘exosome’ pellet [48], suggesting that only a subtype of EV contained this particular molecule. This observation was consistent with a former study showing, after separation of EV subtypes by ‘differential buoyant velocity gradient’ [49], that different miRNA sequences are selectively secreted either in different EVs, or even in non-vesicular nucleosomes. These observations confirm again that ‘exosome’ preparations are not pure, and suggest that not only co-isolated other small EVs, but also co-isolated protein–nucleic acid complexes and other entities may carry part of the functions described for exosomes, especially when these functions are described as mediated by miRNA. Studies analysing the specific nucleic acid types or sequences present in different types of EVs are now starting to be published [30,49–52]. We hope that comprehensively comparing these results will also, in the near future, make it possible to understand the mechanisms and functional consequences of targeting different RNA cargoes to different EV subtypes.

In conclusion of this short text, we would like to direct the reader to important articles and initiatives of the EV field. First, several specific technical difficulties and potential artefacts must be taken into account when studying RNA-related EVs' functions, including the potential contamination by serum-derived components when studying EVs from cultured cells [53]. We encourage any scientist interested in developing new EV-related projects to read a very comprehensive recent overview of all these aspects, written as a follow-up to a dedicated workshop organized in 2015 by the International Society for Extracellular Vesicles (ISEV) [54]. We also suggest that all authors of EV studies take advantage of the EV-TRACK website (http://evtrack.org), developed by an international consortium of EV scientists, to determine whether their study provides sufficient information on the technical aspects of EV isolation and characterization [18]. Finally, it is generally advisable to follow developments of the EV field through ISEV meetings (www.isev.org) and publications in the *Journal of Extracellular Vesicles* (http://www.tandfonline.com/toc/zjev20/current), such as guidelines [14], whose goal is to help the field expand while keeping the highest possible level of technical accuracy and reproducibility.

## Data accessibility

This article has no additional data.
